# Joint Perception of a Shared Object: A Minimalist Perceptual Crossing Experiment

**DOI:** 10.3389/fpsyg.2016.01059

**Published:** 2016-07-12

**Authors:** Loïc Deschamps, Charles Lenay, Katia Rovira, Gabrielle Le Bihan, Dominique Aubert

**Affiliations:** ^1^EA 2223 COSTECH (Connaissance, Organisation et Systèmes Techniques), CRED (Cognitive Research and Enaction Design), Université de Technologie de CompiègneCompiègne, France; ^2^EA 4700 PSY-NCA (Psychologie et Neurosciences de la Cognition et de l’Affectivité), FIACRE (Formation Intentionnelle des Actions, de la Communication et de la Régulation Emotionnelle), Université de RouenMont-Saint-Aignan, France

**Keywords:** perceptual crossing, Participatory Sense-Making (PSM), social cognition, joint attention, minimalism

## Abstract

The minimalist perceptual crossing paradigm has emphasized the essential role of interpersonal dynamics on social understanding. Within the particular case of minimalist interaction, it has been argued that interpersonal processes can constitute social cognition, at least partially, which calls for a paradigm shift in social cognition studies. In this paper, we review several perceptual crossing experiments and their theoretical implications, and propose an original experiment to go beyond strictly dyadic interactions. Whereas past experiments have used objects as distracters of dyadic interaction, our experiment aims at integrating objects themselves as the goal of interpersonal coordination. We asked 24 subjects to participate in a minimalist perceptual crossing experiment where they had to decide, based on their on-line interaction in a one-dimensional digital space, which of the objects they perceived was also perceptible by their partner. The main results suggest that the mutual awareness of a shared object (SO) arises from the quality of sensorimotor coordination between the partners. Indeed, the presence of a SO acts as a simultaneous affordance that attracts and structures individual perceptive activities, giving both partners the opportunity to co-construct a shared world where their respective actions make sense. We discuss our results by way of an enactive account of social cognition, taking the joint perception of a SO as a first step to account for joint attention.

## Introduction

Traditional attempts to explain social cognition have been based on a set of individual internal representations to explain the understanding of the other (e.g., Frith, 2008). In this case, an outside observer usually interprets the observed phenomena, to recognize them as social and to define the intention of the subjects who are interacting (e.g., Gallotti and Frith, 2013). Some alternative approaches, describing an “interactive turn" in the study of social cognition (De Jaegher and Di Paolo, 2007; De Jaegher et al., 2010), try on the contrary to understand these phenomena right from the dynamics of perceptual interactions (e.g., Auvray et al., 2009). It is, however, very difficult to propose explanatory schemes and an experimental situation that produce arguments for a specific discussion between these perspectives.

The minimalist perceptual crossing paradigm that we put into place at the UTC aims to meet this requirement. This experimental setup proposed “the simplest on-line paradigm” (Auvray and Rohde, 2012), which allowed us to closely monitor the co-construction of the interaction process in minimalist social tasks. By oversimplifying the available set of actions and sensory feedback, this paradigm provided the opportunity to precisely analyze the spatiotemporal unfolding of individual activities and collective dynamics (Lenay and Stewart, 2012).

The results of the pioneering study (Lenay et al., 2006; Auvray et al., 2009) and its variants (Lenay and Stewart, 2012; Froese et al., 2014a,b), as we will show, provided an experimental demonstration of the entanglement of individual and collective dimensions in social cognition (De Jaegher and Di Paolo, 2007; Di Paolo and De Jaegher, 2016). Nevertheless, it seemed imperative to us to complete this demonstration, by proposing an experiment that surpasses the frame of dyadic interactions in order to fully illustrate social cognition. We thus will present an original extension of the minimalist perceptual crossing paradigm within the context of triadic/deictic situations (mutual perception of an object, designation, joint attention).

## The Perceptual Crossing Paradigm

**FIGURE 1:**
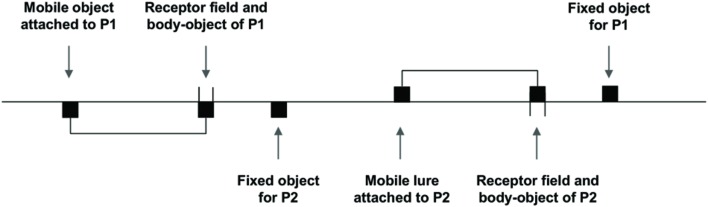
**Schematic illustration of the one-dimensional digital space explored by the participants.** Participant P1 receives a tactile stimulation whenever she encounters either her fixed object, or the receptor field of participant P2, or the mobile object attached to the receptor field of P2 (from Lenay and Stewart, 2012).

The pioneering study of minimalist perceptual crossing was proposed during the last decade (Lenay et al., 2006; Auvray et al., 2009). Two participants were using a technical device in order to interact in a virtual environment. By means of a computer mouse, each participant moves a receptor field laterally in a one-dimensional digital space, and the meeting of this field with any “object” in the environment activates an all-or-none tactile stimulation. Each participant can encounter three kinds of objects, each of which delivers strictly the same stimulation: the body-object moved by the other participant (which is superimposed on her receptor field), a fixed object and a mobile object. It is crucial to note that the mobile object is actually a lure attached to the receptor field of each participant by a rigid virtual link (see **Figure 1**).

Consequently, the mobile object moves exactly like the receptor field to which it is attached, copying all the movements of the latter at a fixed distance. Participants were invited to interact for three 5-min sessions, and they had to click with their mouse whenever they thought the stimulations they were receiving were due to encounters with their partner.

It turns out that participants succeeded in finding each other, and mostly clicked when they actually met. However, a precise analysis revealed that they were unable to differentiate the other participant from the mobile object. Indeed, the ratios between clicks and stimulations showed that participants were as likely to click when the stimulations they received resulted from the encounter with their partner as when they resulted from the encounter with the mobile object. From there, the results can be interpreted in two opposite ways, depending on whether the emphasis in explaining how the task is resolved is put on the interaction process as such (interactionist account) or on the individual processes (individualist account).

The interactionist account focuses on the interaction process to explain the performance (Auvray et al., 2009). As the participants do not actually discriminate their partner from the mobile object, the resolution of the task cannot be attributed to the individual recognition of the other’s intentionality. Instead, while each participant actively searches to interact with each other, the meeting of their perceptual activities gives rise to a kind of “attractor” of the collective dynamics: both participants tend to oscillate one around the other in a more or less coordinated fashion, each perceptual activity being organized by the perceptual activity of the other. By contrast, since the movement of the mobile object is not directed toward the other participant, it can only generate a one-sided coordination that cannot maintain the interaction, as does the perceptual crossing. As a result, the resolution of the task seems to rely on the emergent dynamic property of the interaction process, before any conscious mechanism, simply because it ensures that the participants meet each other more frequently than other sources of stimulations (ibid.). While the collective dynamics is here considered a fully fledged feature in the resolution of the social task, this experiment has led to the controversial idea that interaction can be constitutive of social cognition (De Jaegher et al., 2010).

By contrast, the individualist account, which fits with the classical approach to social cognition (e.g., Frith, 2008), considers that the crucial point of the task remains the individual formation of social judgments. Each individual has merely to detect that the other is moving, and makes their judgment accordingly (Michael and Overgaard, 2012; Overgaard and Michael, 2013). But in order to avoid the mobile object, one should be able to detect that these movements are contingent with one’s own. This was not the case, as the participants did not discriminate the contingent movements of the body-object of the partner from the non-contingent movements of the mobile object. Crucially, as the participants also click when there is no actual interaction (when they meet the mobile object), the interaction process is not involved in (and hence, cannot constitute) social cognition, at least at the level of individual judgment. Therefore, the interactive context is no more than scaffolding for social cognition, by facilitating (in some cases) the individual processes that lead to the decision to click. In a way, social interaction is nothing but informational input among other inputs. In a nutshell, the collective dynamics has no influence as such in the formation of social judgment: social cognition thus remains relegated to a set of individual mechanisms.

## Toward An Entanglement Of Individual And Collective Dimensions

The discussion around these two perspectives has given rise to a set of complementary experiments (for a review, see Auvray and Rohde, 2012). Some original extensions were proposed, for instance where there were no objects, so as to focus on the strictly dyadic interaction alone (e.g., Iizuka et al., 2009; Deschamps et al., 2012). The initial experiment has also been replicated, with the introduction of some crucial variants, such as the multimodal enrichment of the sensory inputs delivered to the participants (Lenay and Stewart, 2012), or as the one-single-click procedure imposed to the participants in a more collaborative version of the initial protocol (Froese et al., 2014a,b). Let’s have a more precise look at these variants.

In the first, a sound was randomly attributed to each of the three objects (Lenay and Stewart, 2012). After each 2-min trial, participants were asked to associate each sound with the corresponding objects. The results show high correct categorizing scores for the three objects (ibid.). Here, the participants’ capacity to distinguish the perceptual crossing dynamics from the encounter with the other objects, in particular with the mobile lures, explained their individual success. The participants could then grasp the interpersonal dynamics with the help provided by the intrinsic sound properties of the objects. If the authors argue in favor of an interactionist approach of social cognition, they also point to the necessary role of the individual, brought to grasp the collective dynamics that she helped to bring out (ibid.). However, a methodological flaw prevents a possible constitutive relation between interaction and social cognition from being determined. In fact, the auto-organization of the perceptual crossing dynamics was determined by a perceptual quality defined *a priori* and above all, outside the interaction itself. In other words, the categorization of the objects was quite explicit on an individual level, but it was not truly constituted collectively (Overgaard and Michael, 2013).

In another variant of the pioneering study, Froese et al. (2014a,b) made the task explicitly collaborative, so that the participants solved the task by an active co-regulation of their perceptual activities. In addition, the participants could only provide one response per trial, and were asked to fill out subjective questionnaires (Perceptual Awareness Scale and Trust Scale) aiming to characterize their experience of the presence of the other participant for each trial (ibid.). In these conditions, the results highlight a very strong propensity for the participants to click on the body-object of their partner, as well as an effective discrimination between this body-object and its attached lure. Interestingly, these clicks were most often carried out together (as opposed to the cases where only one of the two participants in a pair clicked on the other), and in a more or less synchronous manner (even though the participants were not aware of their partner’s click, they both correctly clicked in a very short interval). Furthermore, the clicks performed in these “Joint Success Trials” were correlated with a high degree of turn-taking, as a reflection of an active co-organization of the interaction process.

Under these empirical evidences, the authors claim that the “(…) social judgments were not so much based on an individual recognition of *the other* but rather on a mutually shared recognition of *each other*, i.e., on an interactively shared cognitive process” (Froese et al., 2014a, p. 4, emphasis in original). Of course, one might claim that these results do not really disentangle the possibility of an individualist interpretation. In fact, both participants may have simultaneously recognized the other based on their own judgments (e.g., Michael and Overgaard, 2012). But without any reference to the interaction process as an active part of the performance, a strict individualist interpretation of the clicks may consider their proximity as coincidental and hence, this solution looks hardly convincing. In line with the interpretation of Lenay and Stewart (2012), it seems then imperative to consider together the individual and the collective dimensions in perceptual crossing experiments. For instance, the collective performance observed in the experiment of Froese et al. (2014a,b) was accompanied by a clear experience of the other, on an individual level, as reported in the questionnaires. To sum up, this variation has the merit of demonstrating in a very clear way that the participants’ active co-regulation made possible not only a shared process of mutual recognition, but also a subjective individual experience of the other (ibid.).

To reconcile the individualist and interactionist explanations, hybrid proposals may appear as a good solution. For instance, integrative approaches propose to hold together the processes that stem directly from the dynamics of social interactions (processes that are fast, efficient, stimulus-driven, and relatively inflexible), with the traditional processes of social cognition (processes that are relatively slow, cognitively laborious, and flexible), such as the individual capacity of mindreading (Bohl and van den Bos, 2012). These two types of processes are meant to capture different aspects of social cognition, and are supposed to be simultaneously involved in everyday situations. However, if this integrative approach opens the way for a suitable consideration of the influence of the interaction as such on social cognition, it does not totally solve the problem by creating a dichotomy between two sets of relatively independent processes (for a discussion, see De Jaegher and Di Paolo, 2013; Di Paolo and De Jaegher, 2016). One possible way to account for a functional relationship between the two types of processes may rely on a developmental trajectory between them, through a pluralist approach (Fiebich and Coltheart, 2015). It is here argued that early embodied practices (expressed for instance through bodily coordination) are at the origin of social understanding. If these embodied practices can lead to subsequent “high-level” skills (like mindreading or narrative practices), the point is that they still remain relevant throughout life (e.g., Gallagher and Hutto, 2008; de Bruin and de Haan, 2012). However, the question of how the two types of processes coexist, when they are concurrently available, remains insufficiently addressed so far.

In our view, the true consideration of the individual and collective dimensions in social cognition needs a more genuine paradigm shift. For instance, a non-reductionist and naturalist approach of cognition, as proposed by enaction (Varela et al., 1991; Stewart et al., 2010), leads in fact to consider the “deep entanglement” between interpersonal dynamics and individual capacities for social understanding (Di Paolo and De Jaegher, 2016). This entanglement is fully captured by the concept of *Participatory Sense-Making* (PSM; De Jaegher and Di Paolo, 2007).

To explain PSM, we must start from the definition of two enactive concepts, namely those of *autonomy* and *sense-making*, which are fundamentally intertwined in living organisms (for a full picture of the enactive concepts, see Di Paolo et al., 2010). A network of processes is autonomous if these processes actively participate in the generation and the sustaining of the network that produce them. By this activity, the network generates its own identity and distinguishes itself from its environment, which does not mean that this network does not continually exchange matter and energy with that environment. Crucially, for the self-sustaining of the network, it has to be adaptive, i.e., it must actively regulate its coupling with the environment, and constitute its own values. Sense-making is the ongoing process by which the autonomous systems “cast a web of significance on their world” (ibid., p.4), by continuously generating the meaning of the environment through their own coupling with it. The world is not pre-given but *enacted* through the activity of organisms.

From there, an enactive definition of social interaction is made possible. Social interaction is “a co-regulated coupling between at least two autonomous agents, where (i) the co-regulation and the coupling mutually affect each other, constituting a self-sustaining organization in the domain of relational dynamics, and (ii) the autonomy of the agents involved is not destroyed (although its scope can be augmented or reduced)” (De Jaegher et al., 2010, pp. 442–443). Given this definition, PSM is the interactive facet of sense-making: it designates the process by which individual activities of sense-making come together and merges, bringing out a specific autonomous dynamics, enabling the participants to make sense *of* and *with* the other (De Jaegher and Di Paolo, 2007). In sum, PSM is defined as “the coordination of intentional activity in interaction, whereby individual sense-making processes are affected and new domains of social sense-making can be generated that were not available to each individual on her own” (ibid., p. 497). The unit of analysis of social cognition is thus no longer reduced to the individual, but makes reference to a system as a (self-)organized whole, including the agents involved in the interaction, the process of interaction itself, as well as the context in which these interactions take place. PSM thus proposes a pertinent middle path between a strictly individual (social cognition refers necessarily to individual mechanisms, e.g., Overgaard and Michael, 2013) and a radically interactionist approach (interactive factors are sufficient to account for social cognition, e.g., Auvray et al., 2009). In short, PSM is a suitable framework that focuses on the dynamical relations between the individual and collective dimensions in social understanding (Di Paolo and De Jaegher, 2016).

## Toward Triadic Interactions

Today, in spite of the pertinence of the minimalist perceptual crossing paradigm to illustrate the concept of PSM, an additional step remains to be taken in order to fully account for an enactive approach to social cognition. In fact, social cognition cannot be reduced to dyadic situations, but equally concerns triadic/deictic situations (mutual perception of an object, designation, joint attention). If the concept of PSM theoretically summarizes these specific situations by offering a suitable frame for analyzing the functional links that the individual mechanisms and the collective dynamics maintain as part of the sharing of objects, an experimental support is yet to be provided.

The joint attention phenomenon is paradigmatic for relatively complex triadic situations. It designates the situation where (at least) two participants coordinate their attention so as to share a reference point, whether it is an object, an event or a third person (e.g., Mundy and Newell, 2007). This key skill of social development first requires the awareness that the participants direct an attentional focus on the world, and most importantly, that this focus can be shared. The question of what is exactly shared here, and how this sharing is realized is the main point of numerous contributions in the joint attention literature (for contrastive accounts about joint attention, see the full issue of Seemann, 2012a). In short, we can sketch two main postures: the “jointness” or the “sharing” in joint attention can be considered as an information that has to be processed individually through some internal cognitive operations or as a phenomenon interactively constituted in the ongoing interpersonal coordination (for a discussion, see Seemann, 2012b).

For instance, from an individualist perspective, joint attention would be the result of a reflexive capacity of the individuals involved in the interaction, which makes possible their understanding of the fact that another person has an attentional focus on the same object (Tomasello, 1995). Thus, it is about to associate an intentional attitude (e.g., Gergely and Csibra, 2003) with a capacity to ascribe this attitude to another person (e.g., Tomasello, 1999). Yet this individualist conception leads to minimizing the role of mutual engagement and active co-regulation of behaviors in arriving at social understanding (Gallagher, 2001; Reddy and Morris, 2004; De Jaegher and Di Paolo, 2007). It reduces the phenomena of joint attention to a succession of perceptual *states*, ignoring the dynamic process by which interpersonal coordination takes form (Reddy, 2012). In fact, joint attention designates above all a sensorimotor coordination (Fiebich and Gallagher, 2012; Gallagher, 2012), in other words, a specific sequence of coordinated activities which most often take place in the more global framework of an interactive context, from which it cannot be dissociated (Kidwell and Zimmerman, 2007). It thus unfolds in a mutually available space (Butterworth and Jarrett, 1991), where the very kinetics of the attentional orientation movements are all perceptual cues grasped by the social partners for serving the organization of their own behavior (Moore et al., 1997).

Within this context, the coordination of the attentional focuses can only make sense inside of a pragmatic context that offers, by intermediary of a shared object (SO), a structure to the attentional orientation of the individual and that of her partner. Individually, in fact, the object is an affordance that attracts the attentional focus and organizes the action. In contrast with a strict gibsonian definition of affordance (Gibson, 1979), we define here affordances as local singularities that grasp one’s perceptual activity and organize the subsequent actions as a result of the successive sensory feedbacks. Crucially, the affordance is not directly perceived through a single and isolated stimulation, but is constituted as a function of the particular succession of stimulations over the course of one’s exploratory gestures. As already noted in a recent paper, the three sources of stimulations in perceptual crossing experiments (the fixed object, the mobile object, and the body-object moved by the other participant) can be accounted for in terms of affordances (Froese et al., 2014a): the fixed object provides an opportunity for a spatial determination through a reversible exploratory gesture, while the mobile object only enables the possibility of a dispersed, one-sided and hence, overly unstable interaction. Because of its responsive nature, the body-object moved by the partner affords a globally stable interaction that makes possible an interpersonal coordination (ibid.).

Within the case of triadic interaction, the object of shared attention can be thought of as a kind of “simultaneous affordance” (Marsh et al., 2006): it attracts the attentional focuses in a reciprocal way, each of the participants being able to relocate the SO by their own reversible actions. The combination of this simultaneous affordance with the affordance for a globally stable interaction, provided by the responsive presence of the other, then sets the conditions for PSM (De Jaegher and Di Paolo, 2007). The awareness or the knowledge of the fact that the object of attention is shared is then co-constructed in the meeting of individual activities through the emergence of an autonomous relational dynamics (De Jaegher and Di Paolo, 2007), which is, in our view, an enactive account of joint attention.

As we have already emphasized, this enactive reading of the joint attention phenomenon, which highlights the interdependence of the individual and collective dimensions, suffers from an absence of experimental demonstration. The minimalist perceptual crossing paradigm then arises as the ideal candidate to carry out this mission.

## An Original Perceptual Crossing Experiment

We therefore propose an experiment that aims at studying interpersonal engagement in a shared digital space, going further than strictly dyadic interactions. Here the perceptual crossing of two participants has the mutual perception of an object as the explicit goal: the objects are thus not used as distractors of interpersonal coordination, but are integrated as a mutually intended goal through the process of interaction.

The objective is thus to experimentally demonstrate that interpersonal coordination of perceptual activities (directed toward the other and toward the objects) is a necessary and sufficient condition for joint attention. To force the deployment of this coordination, we will start from a strict minimalist framework, with sensory information reduced to all-or-nothing stimulations (‘Mono’ condition). In addition, we decided to create two other configurations (‘Differentiated’ and ‘Parallelism’ conditions, see below), because we thought that, in the context of interpersonal coordination around SOs, minimal undifferentiated stimulations would lead to major confusion. Indeed, participants cannot perceive an object and the other person at the same time, and cannot try to “point out” an object to her partner without “hiding” it.

## Materials and Methods

### Population

Twenty-four adults, grouped in pairs, participated in this experiment. They were 18- to 25-year-old students, recruited through an ad system at the University of Technology of Compiègne (France). They had no specific knowledge about the device used or the theoretical issues of the experiment. The study protocol followed the tenets of the Declaration of Helsinki of June 1964 (amended during the 64th General Assembly of the World Medical Association in October 2013). All the participants have granted their written informed consent.

### Stimuli and Apparatus

Each participant sat in front of a laptop in one of two quiet adjacent rooms. The laptops were equipped with an optical mouse, a 16-pin tactile stimulator case (namely, two piezoelectric Braille cells corresponding to four columns of four pins) and headphones to receive instructions. Participants were asked to use the mouse with their dominant hand and place the index of their non-dominant hand upon the stimulator case. They were not blindfolded during the experiment, but no information could be seen on their screens. In one of the rooms, a server was present to enable the connection of the two laptops through a Local Area Network.

With their mouse, participants can move a cursor laterally in a one-dimensional digital space 400 pixels long. That space loops on itself, which means that participants are not aware of any boundaries. The cursor is actually a body-object 2-pixels long situated in the middle of a receptor field, 16-pixels long. When the cursor meets at least one colored pixel (from any object of the shared space, including the body-object of the partner), the tactile stimulator case activates in a way that is dependent on the experimental conditions described below.

**FIGURE 2:**
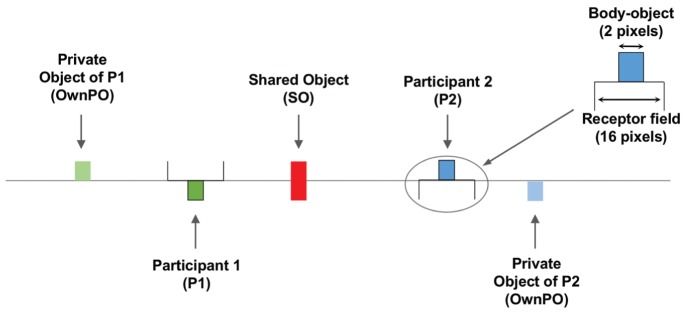
**Schematic illustration of the one-dimensional digital space explored by the participants.** Participant P1 receives a tactile stimulation whenever she encounters either her Private Object, or the Shared Object (SO), or the body-object moved by P2. Note that as illustrated on the right of the figure, the receptor field of P2 is oriented downward: this means that P2 can perceive only the objects (and the part of SO) located below the line, consequently being ignorant of the Private Object of P1.

For each trial, three fixed 2-pixel objects are placed along the one-dimensional space. One of these is jointly perceptible by both participants whereas the two others are private, that is to say that each is respectively perceptible by only one of the two participants (see **Figure 2**). In other words, for a given participant, two objects are perceptible for each trial: a shared one (SO, for Shared Object) and a private one (OwnPO, for Own Private Object). The private object of the partner is thus called OtherPO (for Other Private Object). The position of these objects varies for each trial.

### Design and Procedure

We confronted each pair of participants with the three experimental conditions, always one after the other but in a counterbalanced order for each pair. Each condition is defined as a function of the type of stimulations delivered when the receptor field of a participant meets the fixed objects or the body-object moved by her partner.

In the ‘Mono’ condition, the receptor field is 16 pixels long. When it meets any object of the environment, the 16 pins of the tactile stimulator case are activated in an all-or-none fashion. The stimulations resulting from the meeting of the fixed objects or the body-object of the partner are thus undifferentiated. This condition refers to the basic setup of the minimalist perceptual crossing paradigm, as it was used in previous experiments.

In the ‘Differentiated’ condition, the receptor field is also 16 pixels long. However, this time, when it crosses the body-object of the partner or a fixed object, it activates differentiated stimulations. The meeting of the partner results in the simultaneous activation of the four upper pins, whereas the meeting of any fixed object (whether shared or private) leads to the simultaneous activation of the four lower pins of the stimulator case. With this configuration, participants are able to perceive a fixed object and the partner at the same time: thus, the four upper and the four lower pins are simultaneously activated.

Finally, in the ‘Parallelism’ condition, the receptor field is divided into 4 four adjacent parts of 4 pixels each. Each of these parts refers topologically (or “in parallel”) to each column of pins on the stimulator case. Therefore, when the leftmost part of the receptor field crosses any object of the environment, the four leftmost pins of the stimulator case (the first column) are activated simultaneously. When the second part from the left crosses any object, the second column of pins is activated simultaneously, and so on. As in the ‘Mono’ condition, stimulations are undifferentiated, but here participants are able to perceive a fixed object and their partner at the same time, provided that the distance between the two sources of stimulation lies between 2 and 16 pixels (respectively the length of the objects and that of the receptor field).

Each of these conditions consists in 13 trials: five familiarization trials and eight experimental trials. Familiarization trials are designed so that each participant can acquire a minimal experience of the device before each experimental phase, including the knowledge of the type of stimuli that the objects and the partner may cause, depending on the experimental condition in which they are. In the first familiarization trial, participants are informed that by moving their mouse laterally, they can encounter a single fixed object. No other source of stimulations can be encountered. Participants are asked to explore their environment, to find the object, and crucially, to be attentive to the particular way the stimulator case activates when they find it, because their activation will be different across each condition. In the first condition the participants perform, they are also asked to move in the direction of their choice until they get two successive stimulations. This step consists in indicating to the participants that the two stimulations are not due to the presence of two distinct objects, but that a single object has been encountered twice. Then, the distance between the two stimulations is the total length of the space, which will be identical across the whole experiment. This first trial is not timed, and lasts as long as the experimenter thinks it is necessary.

In the following familiarization trials, participants have the opportunity to interact with their partner during a collaborative four-round mini-game. In these trials, no object is present: the only source of tactile stimulation is then the body-object moved by the partner. As in the first familiarization trial, participants are asked to be attentive to the way the stimulator case activates when they cross the other, as these stimulations can be different from those received when they encountered an object before. Before the mini-game starts, the participants are told that two roles will be alternatively assigned to each of them by way of the headphones. If the participant A is “Guider,” the participant B is “Follower” and vice versa. Guider is informed that he can find a sound at a precise location of the space, but that the Follower cannot hear it. Her objective is to locate this sound, and try to guide her partner there. Follower is informed that the Guider will try to bring him in a particular location of the space. Again, each round is not timed, and stops when the experimenter finds that both participants stop on or oscillate around the sound location. When this is the case, the experimenter starts the next trial, which is identical except that the sound event is moved and that the roles are reversed: the Guider becomes the Follower and vice versa.

Before experimental trials start, we remind the participants that the stimulations they experienced in the familiarization trials when they crossed an object or the body-object of their partner will be identical in the following experimental trials. Participants are asked to collaborate with their partner, interacting tactilely with her, in order to find the SO among their two perceptible objects (SO and OwnPO). Each of these trials lasted 75 s. Individual instructions, given through headphones, indicated the beginning and the end of the trial. Sixty seconds after the trial started, participants were invited to click with their mouse on the SO. The clicks eventually performed before this 15-s time window were not taken into account. After each condition, a 2-min break is proposed, during which the participants cannot interact.

The perceptual trajectories of both participants, including their position in space over time, as well as the exact position of their clicks, were recorded for each trial. When the three experimental conditions were performed, we proceeded to a debriefing where participants could express the difficulties they had and the strategies they used in order to solve the task.

Whatever the experimental condition, we expected the participants to click more often on the SO than on the private ones. Our assumption was that their success would be enabled through the emergence and the stabilization of an interpersonal dynamics around the SO. However, we also expected the experimental conditions to have an impact on this success, notably by modulating the very nature of individual engagement in the environment. Be that as it may, these minimalist conditions would force the spatiotemporal unfolding of perceptual activities and collective dynamics, as it was assumed in previous perceptual crossing experiments. These could be insightful in precisely investigating the nature of interpersonal coordination within the context of triadic interactions.

## Results

### Categories of Clicks

First, we investigated the clicks performed by the participants. We considered four categories of clicks, as a function of the distance between the objects and the receptor field at the time of each click. We assumed a tolerance margin of 32 pixels around each object (corresponding to the length of the receptor field from either side of the objects with at least one pixel in common) to categorize the clicks performed: (i) around the Shared Object (SO clicks); (ii) around the Private Object of the participant (OwnPO clicks); (iii) around the Private Object of the partner (OtherPO clicks); and (iv) at any other location (Empty clicks). Note here that we excluded from the analysis the trials where participants did not click (averaging 3.4% of the trials).

**FIGURE 3:**
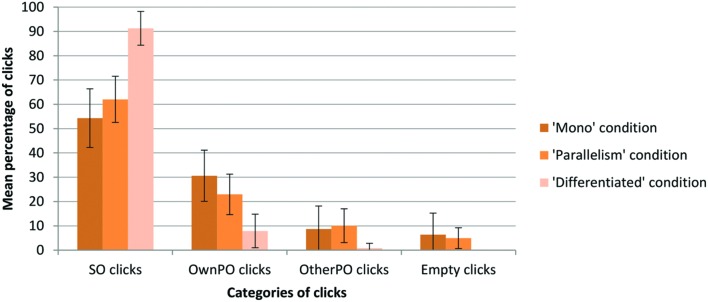
**Mean percentages of clicks assigned to each category as a function of experimental conditions**xs.

We analyzed the average number of clicks assigned to each category for each participant and for each experimental condition (**Figure 3**). As the distribution of this data fitted with a normal curve, we used parametric statistical procedures.

Across all conditions, we observed a mean percentage of SO clicks above the mean percentages of clicks assigned to other categories (**Figure 3**).

A one-way ANOVA showed a significant difference across the categories of clicks for the ‘Mono’ condition [*F*_(3.92)_ = 27.11; *p* < 0.01], for the ‘Differentiated’ condition [*F*_(3.92)_= 452.38; *p* < 0.01], and for the ‘Parallelism’ condition [*F*_(3.92)_= 67.91; *p* < 0.01]. *Post hoc* Tukey’s HSD tests revealed that the mean percentage of SO clicks is greater than the mean percentage of OwnPO clicks (*p* < 0.01), OtherPO clicks (*p* < 0.01) and Empty clicks (*p* < 0.01) for each condition.

### Analysis of Perceptual Trajectories

**FIGURE 4:**
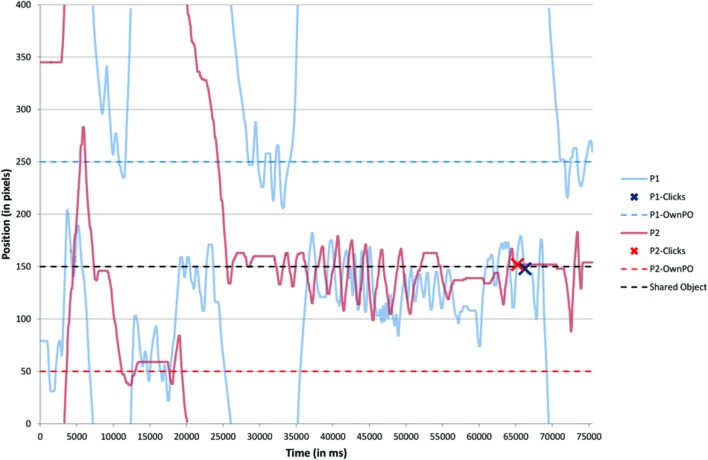
**Time series of an illustrative trial.** The perceptual trajectories of a pair of participants can be spotted across the one-dimensional space (*y*-axis) over the 75 s (*x*-axis) of a given trial (i.e., the second trial of the ‘Mono’ condition). Solid light blue and light red lines plot the position of the two participants (P1 and P2), while the dotted blue and red lines represent the position of their respective private object (P1-OwnPO and P2-OwnPO). The dotted black line plots the position of the Shared Object. In this trial, both participants clicked on the Shared Object (blue and red crosses).

In order to explain these results, we investigated the perceptual trajectories of the participants in each trial (see **Figure 4**).

This analysis sought to explain the clicks performed as a function of what actually happened in each trial. We first carried out a qualitative analysis of these trajectories that we linked to the statements of the participants during the debriefing period of the experiment. We thus identified a set of indicators to characterize the nature and the quality of interpersonal coordination for each category of clicks and for each experimental condition imposed.

Four indicators emerged: (1) the total duration spent by the participants around each object (SO, OwnPO, and OtherPO); (2) the total number of interpersonal stimulations around each object (SO, OwnPO, and OtherPO); (3) the duration of mutual stops around each object (SO, OwnPO, and OtherPO); (4) the total duration of interactive sequences around each object (SO, OwnPO, and OtherPO). For each of these indicators, we aggregated the data of each participant (e.g., by adding the time spent by a given participant around SO and so on) for each trial of the three conditions.

**FIGURE 5:**
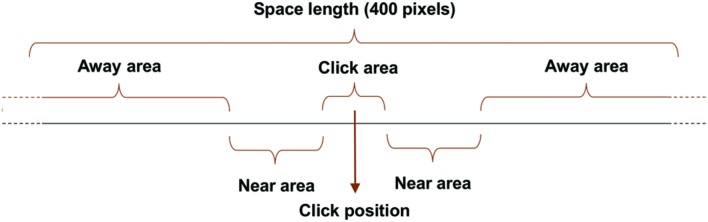
**Schematic illustration of the “Click area,” the “Near Area” and the “Away area,” defined a posteriori as a function of the click position of a participant in a given trial**.

In addition, we split the space into three distinct ranges to investigate the link between this data and the position of the clicks: (i) the “Click Area” extends to a range of 40 pixels around the click position (10% of space length); ii) the “Near area” extends to a range of 60 pixels from either side of the “Click area” (30% of space length), and (iii) the “Away area” extends to a range of 120 pixels from either side of the “Near area” (60% of space length) (**Figure 5**).

As the distribution of this data did not fit with a normal curve, we used non-parametric statistical procedures.

#### Total Duration Spent by the Participants around the Objects

**FIGURE 6:**
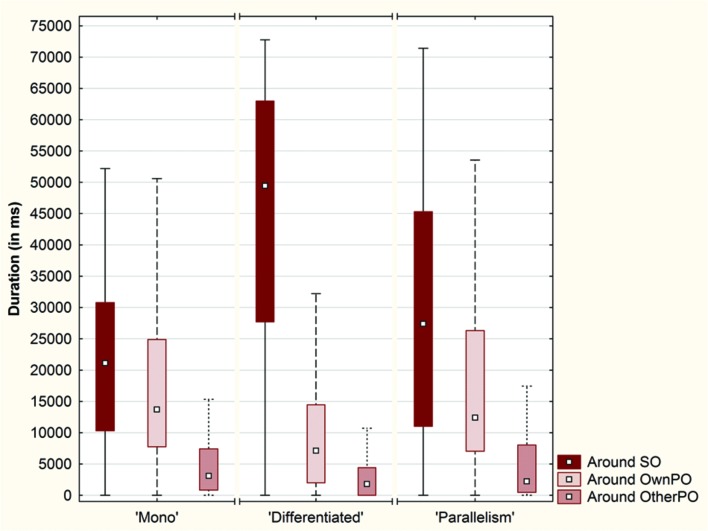
**Box plots of the total duration (in ms) spent by the participants around each object (SO, OwnPO, and OtherPO) over the conditions (Plot: Median, Box: 25–75%, Whisker: Non-Outlier Range)**.

We investigated the total duration spent by the participants in a 40-pixel range around SO, OwnPO, and OtherPO (**Figure 6**).

**FIGURE 7:**
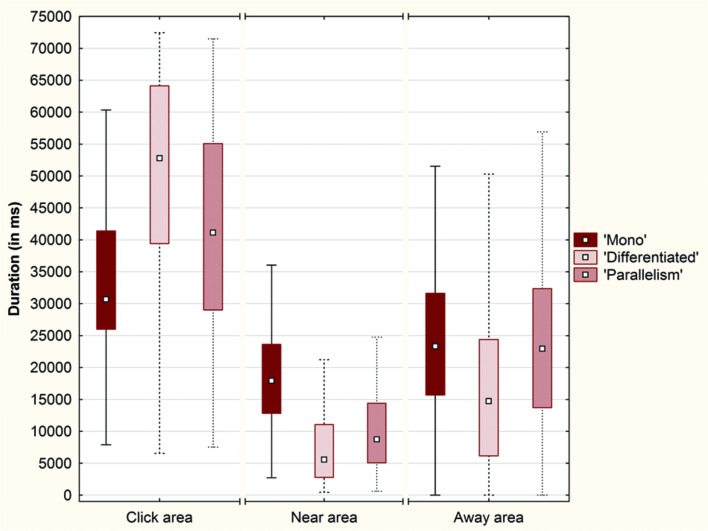
**Box plots of the total duration (in ms) spent by the participants across each condition (‘Mono,’ ‘Differentiated’ and ‘Parallelism’ conditions) over the Click area, the Near area and the Away area (Plot: Median, Box:25–75%, Whisker: Non-Outlier Range)**.

A Friedman ANOVA for dependent samples showed a significant difference in the time spent around the objects across the ‘Mono’ condition (χ^2^= 142.13; *p* < 0.01), across the ‘Differentiated’ condition (χ^2^= 204.29; *p* < 0.01) and across the ‘Parallelism’ condition (χ^2^= 106.55; *p* < 0.01). Wilcoxon matched pairs tests revealed a significantly higher duration spent by the participants around SO than around OwnPO (‘Mono’ condition: *Z* = 2.88; *p* < 0.01; ‘Differentiated’ condition: *Z* = 9.65; *p* < 0.01; ‘Parallelism’ condition: *Z* = 4.07; *p* < 0.01), or around OtherPO (‘Mono’ condition: *Z* = 9.64; *p* < 0.01; ‘Differentiated’ condition: *Z* = 11.56; *p* < 0.01; ‘Parallelism’ condition: *Z* = 8.89; *p* < 0.01). Duration spent around OwnPO was always longer than the duration spent around OtherPO (‘Mono’ condition: *Z* = 8.80, *p* < 0.01; ‘Differentiated’ condition: *Z* = 7.64, *p* < 0.01; ‘Parallelism’ condition: *Z* = 7.34, *p* < 0.01). In addition, we observed that the participants spent more time in the Click area than in the Near and Away areas across each condition (**Figure 7**).

A Friedman ANOVA for dependent samples revealed a significant difference in the duration spent in each interval around the click position across the ‘Mono’ condition (χ^2^= 67.61; *p* < 0.01), across the ‘Differentiated’ condition (χ^2^= 196.88; *p* < 0.01), and across the ‘Parallelism’ condition (χ^2^= 160.54; *p* < 0.01). Wilcoxon matched pairs tests indicated that the duration spent by the participants inside the Click area (10% of space length) was significantly longer than the duration spent inside the Near area (‘Mono’ condition: *Z* = 8.84; *p* < 0.01; ‘Differentiated’ condition: *Z* = 10.85; *p* < 0.01; ‘Parallelism’ condition: *Z* = 10.04; *p* < 0.01) or inside the Away area (‘Mono’ condition: *Z* = 4.80; *p* < 0.01; ‘Differentiated’ condition: *Z* = 9.55; *p* < 0.01; ‘Parallelism’ condition: *Z* = 6.29; *p* < 0.01), although these two areas are respectively 30 and 60% of the space length.

#### Number of Interpersonal Stimulations around the Objects

**FIGURE 8:**
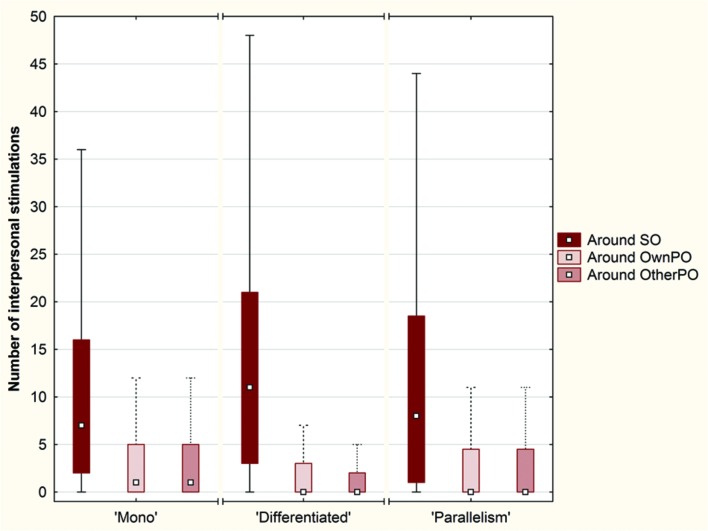
**Box plots of the number of interpersonal stimulations around each object (SO, OwnPO and OtherPO) over the conditions (Plot: Median, Box:25–75%, Whisker: Non-Outlier Range)**.

A second indicator dealt with the number of interpersonal stimulations that occurred, regardless of their duration, in a 40-pixel range around SO, OwnPO, and OtherPO (**Figure 8**).

A Friedman ANOVA for dependent samples showed a significant difference in the number of interpersonal stimul ations around the objects across the ‘Mono’ condition (χ^2^= 59.67; *p* < 0.01), across the ‘Differen tiated’ condition (χ^2^= 145.05; *p* < 0.01), and across the ‘Parallelism’ condition (χ^2^= 48.50; *p* < 0.01). Wilcoxon matched pairs tests revealed that the number of interpersonal stimulations was significantly larger around SO than around OwnPO (‘Mono’ condition: *Z* = 6.07; *p <* 0.01; ‘Differentiated’ condition: *Z* = 9.51; *p* < 0.01; ‘Parallelism’ condition: *Z* = 5.83; *p* < 0.01), and around OtherPO (‘Mono’ condition: *Z* = 6.23; *p* < 0.01; ‘Differentiated’ condition: *Z* = 9.57; *p* < 0.01; ‘Parallelism’ condition: *Z* = 5.91; *p* < 0.01). The difference between OwnPO and OtherPO was not significant for any of the experimental conditions (*p* > 0.05).

**FIGURE 9:**
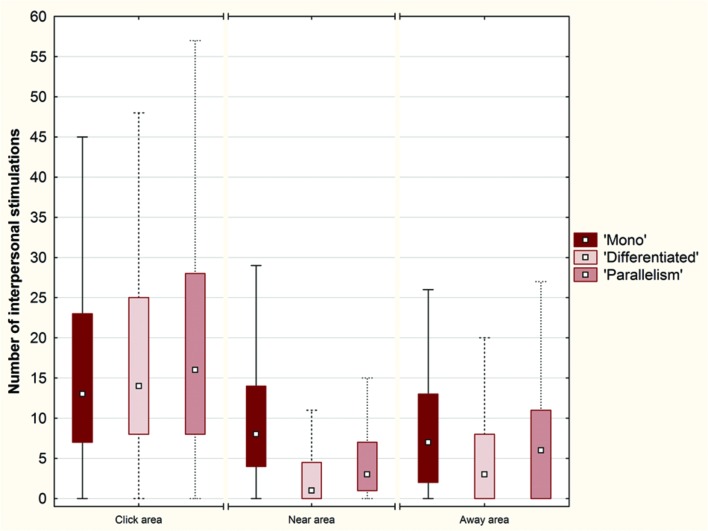
**Box plots of the number of interpersonal stimulations in each condition (‘Mono’, ‘Differentiated’ and ‘Parallelism’ conditions) over the Click area, the Near area and the Away area (Plot: Median, Box:25–75%, Whisker: Non-Outlier Range)**.

In addition, we observed a greater number of interpersonal stimulations in the Click area than in the Near and Away areas across each experimental condition (**Figure 9**).

A Friedman ANOVA for dependent samples indicated a significant difference in the number of interpersonal stimulations occurring in each interval around the click position across the ‘Mono’ condition (χ^2^= 28.62; *p* < 0.01), across the ‘Differentiated’ condition (χ^2^= 139.93; *p* < 0.01), and across the ‘Parallelism’ condition (χ^2^= 69.91; *p* < 0.01). Once again, Wilcoxon matched pairs tests showed a number of interpersonal stimulations significantly larger in the Click area (10% of space length) than in the Near area (‘Mono’ condition: *Z* = 5.28; *p* < 0.01; ‘Differentiated’ condition: *Z* = 10.31; *p* < 0.01; ‘Parallelism’ condition: *Z* = 8.77; *p* < 0.01), and the Away area (‘Mono’ condition: *Z* = 4.19; *p* < 0.01; ‘Differentiated’ condition: *Z* = 7.50; *p* < 0.01; ‘Parallelism’ condition: *Z* = 5.55; *p* < 0.01), although these two areas are respectively 30 and 60% of space length.

#### Duration of Mutual Stops around the Objects

**FIGURE 10:**
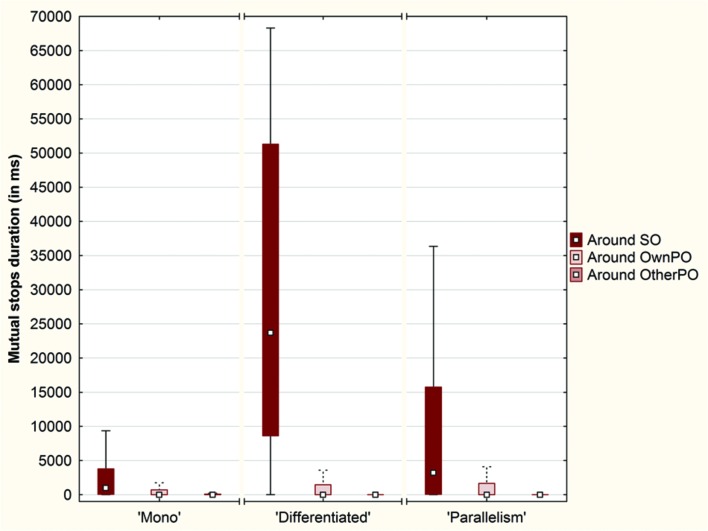
**Box plots of the duration (in ms) of mutual stops around each object (SO, OwnPO and OtherPO) over the conditions (Plot: Median, Box:25–75%, Whisker: Non-Outlier Range)**.

We considered a third indicator: the duration of mutual stops, defined as the total duration where both participants stopped one on the other and maintained a perceptual crossing for at least 2 s (**Figure 10**).

A Friedman ANOVA for dependent samples showed a significant difference in the duration of these mutual stops around the objects across the ‘Mono’ condi tion (χ^2^= 56.83; *p* < 0.01), across the ‘Differentiated’ condition (χ^2^= 178.54; *p* < 0.01), and across the ‘Parallelism’ condition (χ^2^= 43.81; *p* < 0.01). Wilcoxon matched pairs tests revealed that the mutual stops lasted longer if they occurred around SO than around OwnPO (‘Mono’ condition: *Z* = 5.73; *p* < 0.01; ‘Differentiated’ condition: *Z* = 11.09; *p* < 0.01; ‘Parallelism’ condition: *Z* = 6.31; *p* < 0.01), or around OtherPO (‘Mono’ condition: *Z* = 5.70; *p* < 0.01; ‘Differentiated’ condition: *Z* = 11.11; *p* < 0.01; ‘Parallelism’ condition: *Z* = 6.35; *p* < 0.01). However, no difference was found between OwnPO and OtherPO for any of the experimental conditions (*p* > 0.05).

**FIGURE 11:**
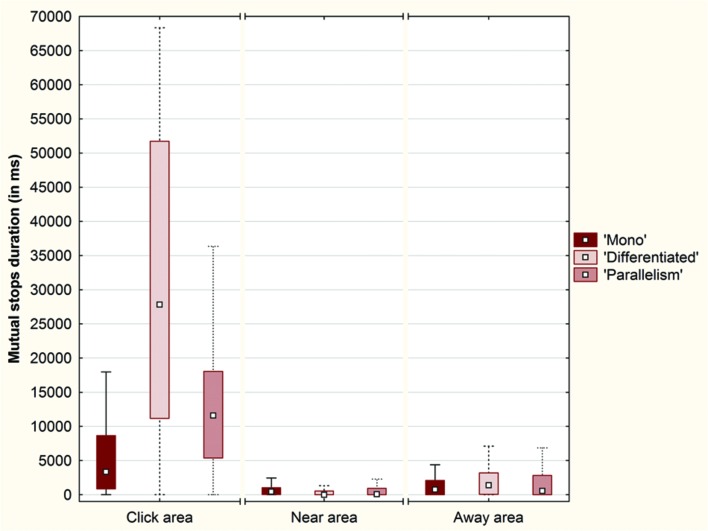
**Box plots of the duration (in ms) of mutual stops across each condition (‘Mono’, ‘Differentiated’ and ‘Parallelism’ conditions) over the Click area, the Near area and the Away area (Plot: Median, Box:25–75%, Whisker: Non-Outlier Range)**.

We brought to light that the participants stopped on each other more frequently in the Click area than in the Near and Away areas across each experimental condition (**Figure 11**).

A Friedman ANOVA for dependent samples showed a significant difference in the duration of mutual stops between the intervals around click position across the ‘Mono’ condi tion (χ^2^= 75.90; *p* < 0.01), across the ‘Differentiated’ condition (χ^2^= 234.83; *p <* 0.01), and across the ‘Parallelism’ condition (χ^2^= 146.49; *p* < 0.01). Wilcoxon matched pairs tests indicated that the duration of mutual stops inside the Click area (10% of the space length) was always longer than those that took place inside the Near area (30% of the space length; ‘Mono’ condition: *Z* = 8.51; *p* < 0.01; ‘Differentiated’ condition: *Z* = 10.90; *p* < 0.01; ‘Parallelism’ condition: *Z* = 9.91; *p* < 0.01) and inside the Away area (60% of the space length; ‘Mono’ condition: *Z* = 6.99; *p* < 0.01; ‘Differentiated’ condition: *Z* = 10.63; *p* < 0.01; ‘Parallelism’ condition: *Z* = 9.29; *p* < 0.01).

#### Duration of Interactive Sequences around the Objects

A final analysis aimed at characterizing the quality of interpersonal coordination between both participants by looking at the duration of the interactive sequences in which they were involved for each trial. We considered a sequence as interactive when it contained at least two interpersonal stimulations and when the gaps between those stimulations did not exceed 2 s. We aggregated the duration of these sequences for each pair of participants in each trial. Note that this indicator did not take into account the duration of the mutual stops when they lasted for more than 2 s.

First of all, it is important to note that the median of the durations of interactive sequences per trial was relatively high in the ‘Mono’ condition (Q1 = 24.22%; Median = 37.62%; Q3 = 44.59%), and in the ‘Parallelism’ condition (Q1 = 25.05%; Median = 34.95%; Q3 = 49.04%). However, the duration of these sequences was lower in the ‘Differentiated’ condition, with a larger interquartile range (Q1 = 12.72%; Median = 24.67%; Q3 = 43.60%).

**FIGURE 12:**
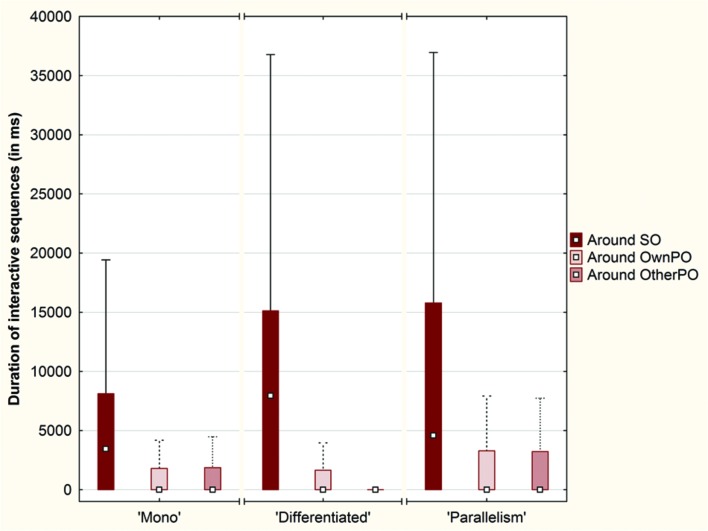
**Box plots of the duration (in ms) of interactive sequences around each object (SO, OwnPO and OtherPO) over the conditions (Plot: Median, Box:25–75%, Whisker: Non-Outlier Range)**.

A Friedman ANOVA for dependent samples concerning the duration of interactive sequences around the objects revealed a significant difference across the ‘Mono’ condition (χ^2^= 45.43; *p* < 0.01), across the ‘Differentiated’ condition (χ^2^= 115.84; *p* < 0.01) and across the ‘Parallelism’ condition (χ^2^= 41.29; *p* < 0.01) (**Figure 12**).

Wilcoxon matched pairs tests showed that the duration of interactive sequences was longer around SO than around OwnPO (‘Mono’ condition: *Z* = 5.53; *p* < 0.01; ‘Differentiated’ condition: *Z* = 8.32; *p* < 0.01; ‘Parallelism’ condition: *Z* = 4.93; *p* < 0.01), and around OtherPO (‘Mono’ condition: *Z* = 5.99; *p* < 0.01; ‘Differentiated’ condition: *Z* = 9.01; *p* < 0.01; ‘Parallelism’ condition: *Z* = 5.82; *p* < 0.01). However, even if we could not conclude there was a difference between OwnPO and OtherPO across the ‘Mono’ and ‘Parallelism’ conditions (*p* > 0.05), the duration of interactive sequences was longer around OwnPO than around OtherPO across the ‘Differentiated’ condition (*Z* = 2.44; *p* = 0.01).

**FIGURE 13:**
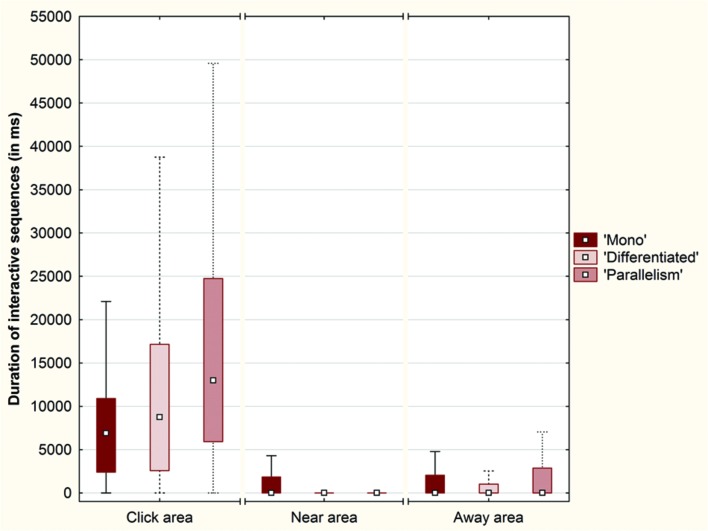
**Box plots of the mean duration (in ms) of interactive sequences per trial across each condition (‘Mono,’ ‘Differentiated’ and ‘Parallelism’ conditions) over the click area, the near area and the away area (Plot: Median, Box:25–75%, Whisker: Non-Outlier Range)**.

Finally, we observed that the interactive sequences were longer in the Click area than in the Near and Away areas across each experimental condition (**Figure 13**).

A Friedman ANOVA for dependent samples showed a significant difference in the duration of interactive sequences as a function of intervals around click positions across the ‘Mono’ condition (χ^2^= 114.66; *p* < 0.01), across the ‘Differentiated’ condition (χ^2^= 162.82; *p* < 0.01), and across the ‘Parallelism’ condition (χ^2^= 169.90; *p* < 0.01). Wilcoxon matched pairs tests indicated that the duration of interactive sequences inside the Click area (10% percent of the space length) was always longer than the duration of interactive sequences inside the Near area (30% of the space length; ‘Mono’ condition: *Z* = 8.74; *p* < 0.01; ‘Differentiated’ condition: *Z* = 9.67; *p* < 0.01; ‘Parallelism’ condition: *Z* = 9.75; *p* < 0.01) and inside the Away area (60% of the space length; ‘Mono’ condition: *Z* = 8.10; *p* < 0.01; ‘Differentiated’ condition: *Z* = 9.02; *p* < 0.01; ‘Parallelism’ condition: *Z* = 9.29; *p* < 0.01).

## Discussion

This original experiment, based on the minimalist perceptual crossing paradigm, is the first to truly go beyond the framework of strictly dyadic interactions. While previous studies used objects as distractors of interpersonal coordination, (e.g., Auvray et al., 2009; Lenay and Stewart, 2012; Froese et al., 2014a,b), we integrate objects here as the explicit goal of this coordination. The challenge of this research is essential because it aims at experimentally illustrating the concept of PSM (e.g., Auvray et al., 2009; Lenay and Stewart, 2012; Froese et al., 2014a,b), as part of an enactive approach to triadic situations (Varela et al., 1991; Stewart et al., 2010). Our main hypothesis consists in the idea that minimal sensorimotor coordination around objects would have the structure of a collective sense-making activity, where the shared or private character of the objects in question would be formed mutually and re-negotiated continuously within the interaction process. Our objective thus consists in trying to understand how, through a dynamics of perceptual crossing reduced to its simplest expression, the participants can perceive and recognize that their partner perceives the object that they perceive. Yet, in the perceptual crossing conditions that we set up, it is difficult to conduct both the task of perceiving an object and the task of perceiving the partner. It is especially difficult in the ‘Mono’ and “Parallelism” conditions, where there are no sensory cues to differentiate the stimulations received in an encounter with the partner from an encounter with any other object.

Generally, the task proposed was a success: whatever the experimental condition, the participants clicked the most often on the SO rather than on the other targets of potential clicks (OwnPO, OtherPO and anywhere else). Despite our initial worry about the inherent difficulty in the “Mono” condition, it has finally proven to succeed quite well, even if we observed fewer clicks on SO than in the other two conditions. Regardless, this result gives evidence of the *individual* recognition of the fact that this object plays an integral part in the shared world, recognition that can only follow from the *collective* dynamics of perceptual interactions. We have here a first approach highlighting the “deep entanglement” between the individual dimension (the decision to click) and the interpersonal dimension (the dynamics of interaction upon which this decision rests) (Di Paolo and De Jaegher, 2016). From this point, the specific analysis of the unfolding of individual activities and collective dynamics must be able to explain more in details the mechanisms leading to this success.

Under the ‘Mono’ condition, to take the most radically minimalist situation, but also under the ‘Parallelism’ condition, there is no perceptual difference between an object and other participants. Yet in the pioneering minimalist perceptual crossing experiment (Lenay et al., 2006; Auvray et al., 2009), the fixed object is interpreted as pertaining to a stable affordance for a reversible perceptual exploration (with possible stops on the object, but always accompanied by a necessary resumption of the movement to maintain the perceptual experience). In other words, the fixed object attracts and organizes the exploration gesture (Lenay et al., 2006; Froese et al., 2014a). This is indeed what we observe here: a *post hoc* analysis reveals that the participants, for the major part of the trials (all conditions taken together), remained around an object perceptible for them (Q1 = 67.45%; Median = 75.94%; Q3 = 82.01%). For all this, we observed that the participants spent significantly more time around SO than around OwnPO, even though the two types of objects have exactly the same objective properties (same size, same form). Here is the first element of importance to explain the success of the task, since the participants spent more time in the area where they finally clicked. The fact that they stay around SO for a long time, compared to the rest of the space, can then account for the high frequency of clicks around this object. How can we explain this result?

The analysis of the indicators concerning the interaction between the participants around the two types of objects allows us to better understand the stronger attraction of SO. The “other” represents a mobile but globally stable affordance for perceptual activities during the perceptual crossing, since, contrary to the fixed objects, it does not hold to its spatial determination (Lenay et al., 2006; Auvray et al., 2009). We note here that, all conditions taken together, the total duration of interpersonal stimulations represents a non-negligible part of the trial time (Q1 = 26.14%; Median = 34.62%; Q3 = 44.63%). The results revealed a significantly higher number of stimulations resulting from perceptual crossing around SO than around the private objects (OwnPO and OtherPO), whatever the experimental condition. If the two types of object equally affect and organize individual perceptual activities, the fact that participants spent more time around SO can thus be explained by the fact that this particular location of the space reciprocally affects and organizes their partner’s activity. In fact, SO proposes a simultaneous affordance (Marsh et al., 2006) that gives the two participants an opportunity to organize their perceptual activities around a shared location of the space, which fosters the interpersonal encounter around that object. In this way, the simultaneous affordance, provided by SO, combines with the specific affordances relative to the encounter of a responsive perceptual activity. From an enactive point of view, we have here the preliminary conditions for the emergence of an autonomous relational dynamics organized around the shared object (De Jaegher and Di Paolo, 2007; De Jaegher et al., 2010).

Furthermore, concurrently with the time spent individually around the different objects, participants tend to click where they most met the other. In fact, to recognize the shared character of an object, that is to say the fact that an object is also perceived by the partner, the participant would have to seek the location where the perception of the other is added to the location of the object. Consistent with this idea, the addition of the two types of affordances – one for an overly stable interaction (with a fixed object) and the other for a globally stable interaction (with the other) – can be seen as a mutually constructed pattern of joint action effects (for details, see Jordan, 2003, 2009). Two additional remarks can be made here.

(i) On the one hand, in the ‘Mono’ condition, perceiving the coincidence of the presence of the other partner and of an object is not easy since this would require knowing how to discriminate between these two presences. Yet the participant only receives one stimulation at a time, the same for the other person or for the object, and thus the sources of the stimulation are mixed. In the “Parallelism” condition, the participant can receive several stimulations at the same time, but they remain undifferentiated (the objects, whether shared or private, and the body-object of the partner deliver the same type of stimulations). Thus, the distinction between those stimulations cannot be made through one single pattern of stimulation, but from a dynamical pattern of sensorial events, arising from the course of an active exploratory gesture. The analysis of the interaction between the two participants goes this way: it revealed that the interactive sequences lasted longer around SO than around the private objects (OwnPO and OtherPO), and that the participants clicked where they interacted the most. It thus seems that the longer these sequences were, the more the participants clicked on SO. The rapid succession of the encounters bears witness to an interpersonal coordination, explicitly or implicitly grasped by the participants as a clue, dynamically and collectively pointing out the shared character of the object.
(ii) On the other hand, the coincidence of the other person and of an object is not sufficient to discriminate SO from the private objects. The participant can just as well find herself in front of her own private object at the same time as being in perceptual interaction with the partner. The object must also be present for the other person, but no participant gains access to the stimulation of the partner to be certain of it. In the ‘differentiated’ condition, the discrimination of the sources of stimulations (partner/fixed objects) is given to the participants beforehand. They thus do not need to make this distinction through their perceptual activity. For all this, we observed that in this condition, the total time of the mutual stops around the shared object was very long. Now, the fact of stopping and maintaining this position shows a certain type of action or at least a behavioral decision that can be attributed to the individual, depending on the context in which she finds herself. Individually, if the participant can then recognize the coincidence of a perceptual crossing with the presence of a fixed object, and if she seeks to maintain it as long as possible, she can suppose that the partner would also maintain the perceptual crossing if she recognizes that coincidence. In a certain manner, we could then conclude that the participant individually passes a social judgment on the basis of objective perceptual clues (e.g., Michael and Overgaard, 2012; Overgaard and Michael, 2013).

However, this partial interpretation does not take into account the fundamental reason for which this type of information can occur. No matter the condition, SO is different from the other objects because the participant is most often present in its area, not only because she encounters the partner and an object, but also because the partner reciprocally encounters at the same location an object and an affordance for interpersonal coordination. In the framework of a joint action, each participant maintains her own activity around this object because she is simultaneously subjected to the proper effect of an object (i.e., an affordance for a reversible exploratory gesture) and to the proper effect of the presence of her partner (i.e., an affordance for a globally stable interaction) (e.g., Jordan, 2003, 2009). The resolution of the task seems to result from a shared process, the presence of each being explained by the presence of the partner and of an object.

The acknowledgment of the shared character of an object rests on the reciprocal capacity of the participants to make sense of their respective actions as a function of the objects present. We can see in this statement the deep entanglement between individual and collective dimensions, as proposed by PSM: the recognition of the fact that an object in the environment is shared arises from the relational dynamics, as an autonomous process that influences the individual contributions and allows the participant to give them meaning (De Jaegher and Di Paolo, 2007; De Jaegher et al., 2010). Both collective and individual dimensions play a role in the resolution of the task, and a focus on one of these two sides cannot fully explained the results.

The autonomous character of this process, characterizing the influence of the collective dynamics on the individual decision to click, is made apparent by one of the mistakes made by some of the participants. It is in fact striking to see that the cases of trials where the participant clicked on the empty point corresponding to the position of the private object of the partner (OtherPO) is relatively frequent in the ‘Mono’ (8.72%) and the ‘Parallelism’ (10.05%) conditions, contrary to the ‘Differentiated’ condition, where only one trial out of 96 led to this situation. In that concrete case, the mere presence of the partner around her private object was sufficient for the participant to believe in the presence of an object. For that matter, it is interesting to note that the probability that the partner clicked on her private object at the same time as the participant clicked on this position is very high, in all the conditions: ‘Mono’ (71.42%), ‘Parallelism’ (85.71%) and ‘Differentiated’ (100%). From the partner’s point of view, however, we have to recognize that all the conditions were met to justify the decision to click on this object (notably, maintaining the other’s activity around a perceptible object). The participants were thus caught up in an interaction dynamics that brought about a co-construction of sense, even if that sense was here erroneous, showing once again the influence of the interaction process on the individual decision.

In this experiment, the participants coordinate their attention so as to recognize that an object of the environment is a shared point of reference. Here it is a question of an operational definition of joint attention (Mundy and Newell, 2007; Seemann, 2012a). Our results demonstrate that this skill can be realized minimally through the sensorimotor coordination of the participants (Fiebich and Gallagher, 2012; Gallagher, 2012). What is more, we have defended here an enactive interpretation of joint attention as a process revealing both individual and collective involvement (Reddy and Morris, 2004; Reddy, 2012). Our results highlight the entanglement of these two levels (Di Paolo and De Jaegher, 2016), and make this original experiment of minimalist perceptual crossing a new demonstration of PSM (De Jaegher and Di Paolo, 2007), this time as part of triadic interactions. Our results then question the classical approach of joint attention as deeply individualistic (Tomasello, 1995, 1999), interpreted at best as a coincidence of individual processing (Gallotti and Frith, 2013) and having as cues objective behaviors, certainly dynamic (Moore et al., 1997), yet disconnected from their interactional context (Kidwell and Zimmerman, 2007).

To be sure, this enactive approach does not take into account more complex situations of joint attention, notably when one of the participants actively directs her partner’s attentional focus toward an object of the environment (intentional joint attention, see Fiebich and Gallagher, 2012). Nor when the point of reference to share is precisely not an object available in the actuality of the interaction (for example in the case of the attentional coordination around an absent object, most frequently carried out using language).

In spite of the apparent stability of the strategies observed in function of the experimental conditions, one possible limit can be highlighted. In fact, in this experiment, the participants carried out the three experimental conditions. If we have offset the order of execution of the conditions, so as to neutralize a possible effect of the order, it is possible that the strategies used in one condition were influenced by the preceding experimental conditions. For example, the fact of beginning with the ‘differentiated’ condition could have led to a type of strategy that showed it to be incompatible with the succeeding conditions, which could have influenced the results. Future experiments will then need to test a possible interaction effect of the experimental conditions on the individual and collective strategies. Furthermore, it would be interesting to separate the experimental conditions according to independent experimental groups. In this way, we would be able to propose more trials, so as to promote the appropriation of the system by the participants and thus permit better individual and collective performances.

## Conclusion

In this research, we proposed an extension of the perceptual crossing paradigm in such a way as to go beyond the framework of dyadic interactions. Our results supplement the enactive approach to social cognition, by demonstrating in an empirical way that the interaction dynamics can also support the recognition of a common world. Our results notably underline a deep entanglement between the individual mechanisms implicated in the performance, and the interaction dynamics as an autonomous process (Di Paolo and De Jaegher, 2016). From this point of view, we have illustrated the concept of PSM (De Jaegher and Di Paolo, 2007) within triadic situations, highlighting a minimalist and sensorimotor approach to joint attention.

## Author Contributions

LD was involved in all the steps of the present work. CL and KR participated in the conception/design of the study, the interpretation of results, the drafting and the revising, and in the final approval of the paper. GL participated in the acquisition, analysis and interpretation of the data, and in the final approval of the paper. DA participated in the conception/design of the study, the acquisition of data, the final approval of the paper, and developed all the software used in the experiment.

## Conflict of Interest Statement

The authors declare that the research was conducted in the absence of any commercial or financial relationships that could be construed as a potential conflict of interest.

## References

[B1] Auvray M. Lenay C. Stewart J. (2009). Perceptual interactions in a minimalist virtual environment. *New Ideas Psychol.* 27 32–47. 10.1016/j.newideapsych.2007.12.002

[B2] Auvray M. Rohde M. (2012). Perceptual crossing: the simplest online paradigm. *Front. Hum. Neurosci.* 6:181 10.3389/fnhum.2012.00181 PMC3377933 22723776

[B3] Bohl V. van den Bos W. (2012). Toward an integrative account of social cognition: marrying theory of mind and interactionism to study the interplay of Type 1 and Type 2 processes. *Front. Hum. Neurosci.* 6:274 10.3389/fnhum.2012.00274 PMC3468956 23087631

[B4] Butterworth G. Jarrett N. (1991). What minds have in common is space: spatial mechanisms serving joint visual attention in infancy. *Br. J. Dev. Psychol.* 9 55–72. 10.1111/j.2044-835X.1991.tb00862.x

[B5] de Bruin L. de Haan S. (2012). Enactivism and social cognition: in search of the whole story. *J. Cogn. Semiotics* 4 225–250. 10.1515/cogsem.2009.4.1.225

[B6] De Jaegher H. Di Paolo E. (2007). Participatory sens-making. An enactive approach to social cognition. *Phenomenol. Cogn. Sci.* 6 485–507. 10.1007/s11097-007-9076-9

[B7] De Jaegher H. Di Paolo E. (2013). Enactivism is not interactionism. *Front. Hum. Neurosci.* 6:345 10.3389/fnhum.2012.00345 PMC3539800 23316153

[B8] De Jaegher H. Di Paolo E. Gallagher S. (2010). Can social interaction constitute social cognition? *Trends Cogn. Sci*. 14 441–447. 10.1016/j.tics.2010.06.009 20674467

[B9] Deschamps L. Le Bihan G. Lenay C. Rovira K. Stewart J. Aubert D. (2012). “Interpersonal recognition through mediated tactile interaction,” in *Proceedings of IEEE Haptics Symposium 2012*, Vancouver, BC, 239–245. 10.1109/HAPTIC.2012.6183797

[B10] Di Paolo E. De Jaegher H. (2016). “Neither individualistic, nor interactionist,” in *Embodiement, Enaction, and Culture*, eds Durt C. Fuchs T. Tewes C. (Cambridge, MA: MIT Press).

[B11] Di Paolo E. De Jaegher H. Rohde M. (2010). “Horizons for the enactive mind: values, social interaction, and play,” in *Enaction Towards a New Paradigm for Cognitive Science*, eds Stewart J. Gapenne O. Di Paolo E. (Cambridge, MA: MIT Press), 33–87.

[B12] Fiebich A. Coltheart M. (2015). Various ways to understand other minds: towards a pluralistic approach to the explanation of social understanding. *Mind Lang.* 30 235–258. 10.1111/mila.12079

[B13] Fiebich A. Gallagher S. (2012). Joint attention in joint action. *Philos. Psychol.* 26 571–587. 10.1080/09515089.2012.690176

[B14] Frith C. D. (2008). Social cognition. *Philos. Trans. R. Soc. B Biol. Sci.* 363 2033–2039. 10.1098/rstb.2008.0005 PMC2375957 18292063

[B15] Froese T. Iizuka H. Ikegami T. (2014a). Embodied social interaction constitutes social cognition in pairs of humans: a minimalist virtual reality experiment. *Sci. Rep.* 4:3672 10.1038/srep03672 PMC3890942 24419102

[B16] Froese T. Iizuka H. Ikegami T. (2014b). Using minimal human-computer interfaces for studying the interactive development of social awareness. *Front. Psychol.* 5:1061 10.3389/fpsyg.2014.01061 PMC4176033 25309490

[B17] Gallagher S. (2001). The practice of mind. Theory, simulation or primary interaction? *J. Consci. Stud.* 8 83–108.

[B18] Gallagher S. (2012). “Interactive coordination in joint attention,” in *Joint Attention: New Developments in Psychology, Philosophy of Mind, and Social Neuroscience*, ed. Seeman A. (Cambridge, MA: MIT Press), 293–306.

[B19] Gallagher S. Hutto D. (2008). “Understanding others through primary interaction and narrative practice,” in *The Shared Mind: Perspectives on Intersubjectivity* Vol. 12 *Converging Evidence in Language and Communication Research*, eds Zlatev J. Racine T. P. Sinha C. Itkonen E. (Amsterdam: John Benjamins Publishing Company), 17–38.

[B20] Gallotti M. Frith C. D. (2013). Social cognition in the we-mode. *Trends Cogn. Sci.* 17 160–165. 10.1016/j.tics.2013.02.002 23499335

[B21] Gergely G. Csibra G. (2003). Teleological reasoning in infancy: the naıve theory of rational action. *Trends Cogn. Sci.* 7 287–292. 1016/S1364-6613(03)00128-1 12860186 10.1016/s1364-6613(03)00128-1

[B22] Gibson J. J. (1979). *The Ecological Approach to Visual Perception.* Boston, MA: Houghton Miﬄin.

[B23] Iizuka H. Ando H. Maeda T. (2009). “The anticipation of human behavior using ‘parasitic humanoid’,” in *Human-Computer Interaction. Ambient, Ubiquitous and Intelligent Interaction*, ed. Jacko J. A. (Berlin: Springer), 284–293. 10.1007/978-3-642-02580-8_31

[B24] Jordan J. S. (2003). Emergence of self and other in perception and action. *Conscious. Cogn.* 12 633–646. 10.1016/S1053-8100(03)00075-8 14656506

[B25] Jordan J. S. (2009). Forward-looking aspects of perception-action coupling as a basis for embodied communication. *Discourse Process.* 46 127–144. 10.1080/01638530902728959

[B26] Kidwell M. Zimmerman D. H. (2007). Joint attention as action. *J. Pragmat.* 39 592–611. 10.1016/j.pragma.2006.07.012

[B27] Lenay C. Auvray M. Sebbah F. Stewart J. (2006). “Perception of an intentional subject: an enactive approach,” in *Proceedings of the Third International Conference on Enactive Interfaces*, Montpellier, 37–38.

[B28] Lenay C. Stewart J. (2012). Minimalist approach to perceptual interactions. *Front. Hum. Neurosci.* 6:98 10.3389/fnhum.2012.00098 PMC3348522 22582041

[B29] Marsh K. L. Richardson M. J. Baron R. M. Schmidt R. C. (2006). Contrasting approaches to perceiving and acting with others. *Ecol. Psychol.* 18 1–38. 10.1207/s15326969eco1801_1

[B30] Michael J. Overgaard S. (2012). Interaction and social cognition: a comment on Auvray et al.’s perceptual crossing paradigm. *New Ideas Psychol.* 30 296–299. 10.1016/j.newideapsych.2012.02.001

[B31] Moore C. Angelopoulos M. Bennett P. (1997). The role of movement in the development of joint visual attention. *Infant Behav. Dev.* 20 83–92. 10.1016/S0163-6383(97)90063-1

[B32] Mundy P. Newell L. (2007). Attention, joint attention, and social cognition. *Curr. Dir. Psychol. Sci.* 16 269–274. 10.1111/j.1467-8721.2007.00518.x 19343102 PMC2663908

[B33] Overgaard S. Michael J. (2013). The interactive turn in social cognition research: a critique. *Philos. Psychol.* 28 160–183. 10.1080/09515089.2013.827109

[B34] Reddy V. (2012). “A gaze at grips with me,” in *Joint Attention: New Developments in Psychology, Philosophy of Mind, and Social Neuroscience*, ed. Seeman A. (Cambridge, MA: MIT Press), 137–157.

[B35] Reddy V. Morris P. (2004). Participants don’t need theories knowing minds in engagement. *Theory Psychol.* 14 647–665. 10.1177/0959354304046177

[B36] Seemann A. (ed.) (2012a). *Joint Attention: New Developments in Psychology, Philosophy of Mind, and Social Neuroscience.* Cambridge, MA: MIT Press.

[B37] Seemann A. (ed.) (2012b). “Joint attention: towards a relational account,” in *Joint Attention: New Developments in Psychology, Philosophy of Mind, and Social Neuroscience*, (Cambridge, MA: MIT Press), 183–202.

[B38] Stewart J. Gapenne O. Di Paolo E. (Eds) (2010). *Enaction: Toward a New Paradigm for Cognitive Science.* Cambridge, MA: MIT Press 10.7551/mitpress/9780262014601.001.0001

[B39] Tomasello M. (1995). “Joint attention as social cognition,” in *Joint Attention: Its Origins and Role in Development*, eds Moore C. Dunham P. J. (Hillsdale, NJ: Lawrence Erlbaum Associates, Inc.), 103–130.

[B40] Tomasello M. (1999). *The Cultural Origins of Human Cognition.* New York, NY: Harvard University Press.

[B41] Varela F. J. Thompson E. Rosch E. (1991). *The Embodied Mind: Cognitive Science and Human Experience*, 6th Edn Cambridge, MA: MIT Press.

